# Efficient Construction of Homozygous Diploid Strains Identifies Genes Required for the Hyper-Filamentous Phenotype in *Saccharomyces cerevisiae*

**DOI:** 10.1371/journal.pone.0026584

**Published:** 2011-10-21

**Authors:** Kentaro Furukawa, Takako Furukawa, Stefan Hohmann

**Affiliations:** Department of Cell and Molecular Biology/Microbiology, University of Gothenburg, Gothenburg, Sweden; University of Calgary, Canada

## Abstract

Yeast cells undergo diploid-specific developments such as spore formation via meiosis and pseudohyphal development under certain nutrient-limited conditions. Studies on these aspects require homozygous diploid mutants, which are generally constructed by crossing strains of opposite mating-type with the same genetic mutation. So far, there has been no direct way to generate and select diploids from haploid cells. Here, we developed a method for efficient construction of homozygous diploids using a *PGAL1-HO* gene (galactose-inducible mating-type switch) and a *PSTE18-URA3* gene (counter selection marker for diploids). Diploids are generated by transient induction of the HO endonuclease, which is followed by mating of part of the haploid population. Since the *STE18* promoter is repressed in diploids, diploids carrying *PSTE18-URA3* can be selected on 5-fluoroorotic acid (5-FOA) plates where the uracil prototrophic haploids cannot grow. To demonstrate that this method is useful for genetic studies, we screened suppressor mutations of the complex colony morphology, strong agar invasion and/or hyper-filamentous growth caused by lack of the Hog1 MAPK in the diploid Σ1278b strain background. Following this approach, we identified 49 suppressor mutations. Those include well-known positive regulator genes for filamentous growth signaling pathways, genes involved in mitochondrial function, DNA damage checkpoint, chromatin remodeling, and cell cycle, and also previously uncharacterized genes. Our results indicate that combinatorial use of the *PGAL1-HO* and *PSTE18-URA3* genes is suitable to efficiently construct and select diploids and that this approach is useful for genetic studies especially when combined with large-scale screening.

## Introduction

Over the last decades genetic studies using the budding yeast *Saccharomyces cerevisiae* have led to discovery of a variety of cellular signaling components as well as many other fundamental cellular processes. One of the advantages of yeast genetics is that it is straightforward to isolate desired mutant strains and identify the underlying mutations. In principle, such genetic approaches can be applied only in the haploid backgrounds because it is difficult to isolate recessive mutations in diploids due to complementation of the phenotype by the second copy of the gene. This becomes an issue when mutant strains defective in diploid-specific developments such as meiosis, sporulation, spore germination, bipolar budding pattern, and pseudohyphal development need to be isolated. Although a yeast homozygous knockout library of the S288C background is available [1], this genetic background has lost some of these specific phenotypes and hence those are commonly studied in other strain backgrounds. Therefore, a method for efficient construction of homozygous double mutants is required.

**Figure 1:**
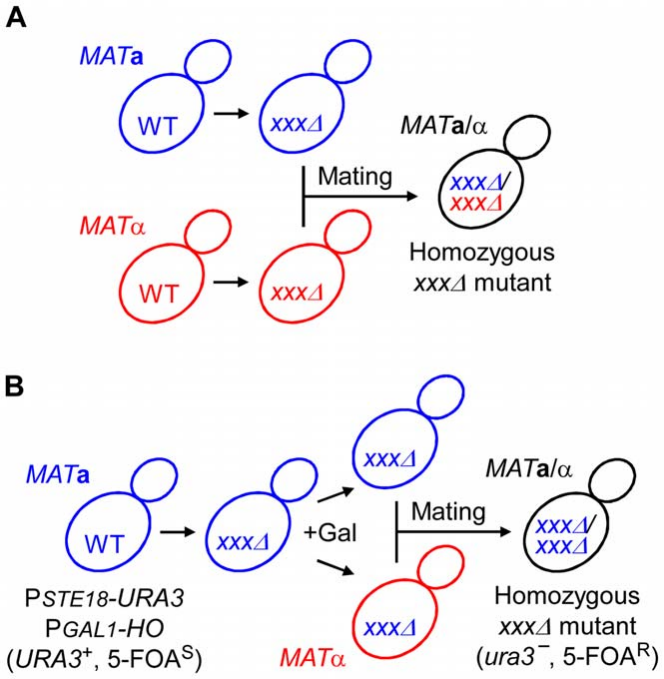
Strategy for construction of homozygous diploid strains. (A) The traditional method requires individual construction of *MAT* **a** and *MAT* **α** haploid strains carrying different selection markers (linked to mutations or each strain). The diploid strains can be selected on plates lacking nutrients or containing antibiotics. (B) The new method proposed in this study generates homozygous diploids from a single haploid strain by subsequent use of the *PGAL1-HO* (galactose-inducible mating-type switch) and *PSTE18-URA3* (counter selection marker for diploids) genes. The diploid strains are selected on plates containing 5-FOA, where non-mated haploid strains cannot grow.

The yeast sexual cell types are designated **a** and **α**, which are conferred by the *MAT* **a** and *MAT* **α** alleles of the *M*ating-*T*ype *L*ocus (*MAT*), respectively [2]. In general, homozygous diploid mutant strains (i.e. *MAT* **a** */* **α** *xxxΔ/xxxΔ*) are constructed by crossing strains of the opposite mating-type, which need to be constructed individually. When the two haploids have different prototrophic or antibiotic resistance markers, the diploids can be easily selected on plates lacking both nutrients or containing both antibiotics because auxotrophy or antibiotic sensitivity are complemented by each genotype (Figure 1A). The HO endonuclease, which mediates mating-type switch, can be used to obtain diploids via mating of *MAT* **a** and *MAT* **α** cells within colonies [3]. Alternatively, zygotes (dumbbell-shaped cells) can be isolated by micromanipulation during conjugation of two cells. However, these methods are unsuitable for large-scale analysis. Thus, there has been no easy way to construct and select diploid strains from single haploids at high throughput so far.

Decreasing gene dosage by RNAi (restored by introducing Dicer and Argonaute from *S. castellii*) [4] or by haplo-insufficiency (heterozygous mutant) [5] may be useful for studying diploid-specific developments. Since these methods do not completely abolish gene function and consequently might give false negative or positive results, an efficient method to create homozygous deletion mutants is desired. In this study, we present an efficient method for construction of diploid strains using a galactose-inducible mating-type switch gene (*PGAL1-HO*) and a counter selection marker gene (*PSTE18-URA3*).

We applied our method to the study of yeast morphological developments. In the *S. cerevisiae* Σ1278b background, diploid cells develop pseudohyphae (filamentous growth) under nitrogen starvation. Since filamentous growth is essential for virulence of yeast pathogens such as *Candida albicans* [6], discovery of positive regulators for filamentous growth using *S. cerevisiae* as a model organism can contribute to understanding common conserved mechanisms. The high-osmolarity glycerol (HOG) response MAPK pathway, which plays a central role in osmoadaptation [7], [8], negatively regulates filamentous growth and deletion of the *HOG1* MAPK gene leads to hyper-filamentous phenotype even under nutrient-rich conditions [9], [10]. In order to identify positive regulators essential for filamentous growth, we performed large-scale construction of homozygous double mutants in theΣ1278b *hog1Δ/hog1Δ* background. The screen identified 49 suppressor mutations, showing that our method is useful for genetic study.

## Results and Discussion

### Efficient Construction of Yeast Homozygous Diploid Strains

Our strategy for construction of homozygous diploid cells is shown in Figure 1B. As a host strain, we used either a *MAT* **a** or *MAT* **α** haploid strain carrying; i) *PSTE18-URA3*, which expresses the *URA3* gene under the control of haploid specific *STE18* (G-protein γ subunit) promoter [11] and ii) *PGAL1-HO*, which expresses the HO endonuclease under the control of *GAL1* promoter (repressed by glucose and induced by galactose [12]). Once the host strain is transiently incubated on galactose plates (*HO* induction), the mating-type switch (*MAT* **a**→*MAT* **α** or *MAT* **α**→*MAT* **a**) occurs and consequently diploid cells are formed by mating of *MAT* **a** and *MAT* **α** cells within colonies. Following short induction times of *HO* (<12 hours), the colonies contain three cell types, *MAT* **a**, *MAT* **α**, and *MAT* **a** */* **α**. Our strategy can select diploids by counter selection using 5-FOA, where haploids cannot grow because Ura3 (orotidine-5′-phosphate decarboxylase) converts 5-FOA into a toxic compound [13], while diploids are resistant to 5-FOA because *URA3* is not expressed. Leaky expression of *HO* does not matter as long as the host strain is maintained on plates lacking uracil.

**Figure 2:**
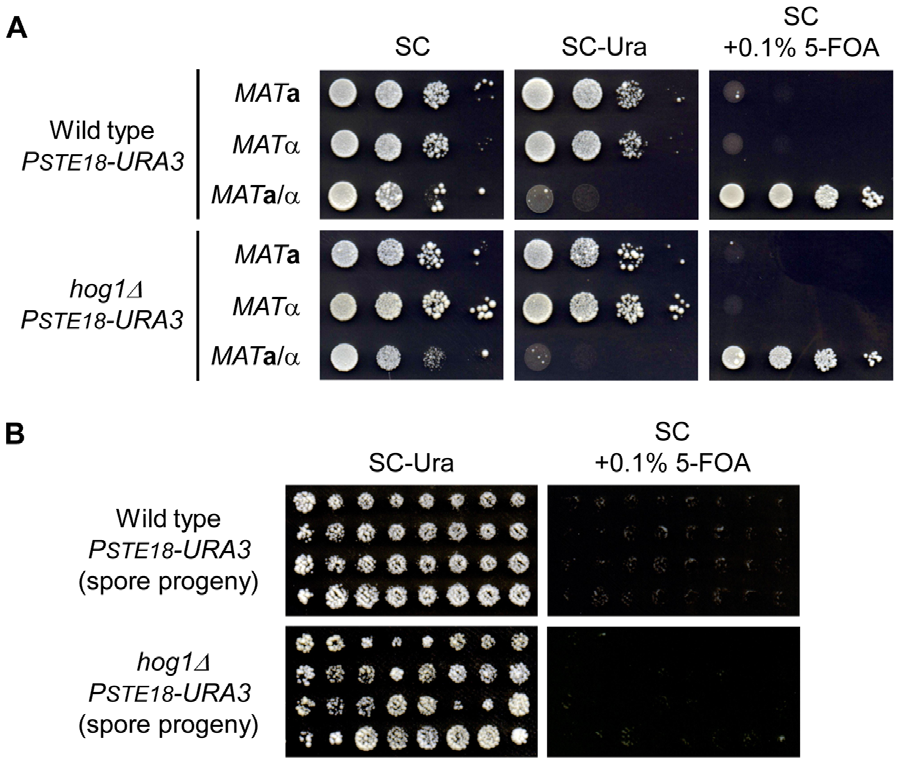
Effect of the *PSTE18-URA3* gene on growth of haploids and diploids. (A) The haploid and diploid *PSTE18-URA3* strains display opposite growth phenotypes on plates lacking uracil or containing 5-FOA. The strains were grown for 2–3 days at 30°C. (B) The growth phenotype of the diploid *PSTE18-URA3* strain can revert to that of haploid after sporulation. The indicated diploid strains were sporulated, tetrads were dissected and spore progeny was grown on YPD plate for 3 days at 30°C. Then, the cells were replicated on the indicated plates and grown for 2–3 days at 30°C.

First, we investigated whether the *PSTE18-URA3* gene allows selecting for diploid cells. We constructed wild-type *PSTE18-URA3* and *hog1Δ PSTE18-URA3* strains in the three cell types (*MAT* **a**, *MAT* **α**, and *MAT* **a** */* **α**) and grew them on SC plates lacking uracil or containing 0.1% 5-FOA. As shown in Figure 2A, the haploid and diploid *PSTE18-URA3* strains showed the expected phenotypes, i.e. the haploid strains were uracil prototrophic and 5-FOA sensitive, and the diploid strains were uracil auxotrophic and 5-FOA resistant. Moreover, the haploid cells that germinated from spore progeny of the diploid *PSTE18-URA3* strains displayed uracil prototrophy and 5-FOA sensitivity (Figure 2B). Taken together, these results demonstrate that the *PSTE18-URA3* gene can be used as a diploid selection marker.

Next, we determined the efficiency of construction of diploids by our strategy. The wild-type *PSTE18-URA3* and *hog1Δ PSTE18-URA3* strains (*MAT* **a** and *MAT* **α**) carrying pJH283 (*PGAL1-HO*) were grown overnight on galactose plate, and then cells were restreaked on SC plate containing 0.1% 5-FOA. The single colonies obtained on the 5-FOA plate were analyzed by two methods: i) observation of pseudohyphal growth on SLAD plate, and ii) determination of mating type by PCR. All of the single colonies analyzed (10 colonies for each strain) showed diploid specific patterns: i) strongly enhanced pseudohyphal growth (data not shown), and ii) two PCR bands corresponding to diploids (Figure S1). These results indicate that our method is highly efficient for construction of diploid strains.

### Screening Suppressor Mutations of Enhanced Morphological Developments of *hog1Δ/hog1Δ*

To demonstrate that our strategy for construction of diploid strains is useful for genetic studies of diploid-specific developments, we applied it to yeast filamentous growth in the Σ1278b background. Transposon insertion mediated-random mutagenesis and -systematic gene disruption have previously been employed to dissect the genetic bases of filamentous growth [14], [15]. However, these studies used haploid strain backgrounds in which filamentous growth was ectopically induced by an extra copy of the opposite mating-type locus and the *PHD1* gene (transcriptional activator for filamentation) or by adding 1% butanol to the growth medium. In addition, however, homozygous diploid strains must be used to analyze the genetics of filamentous growth. We have recently reported that hyperosmotic stress inhibits all of the yeast morphological developments and that the Hog1 MAPK is a central negative regulator of these developments [10]. Moreover, the effect of *HOG1* deletion is reflected in diploids more clearly than in haploids [16]. Therefore, suppressor mutations of the enhanced morphological developments of *hog1Δ/hog1Δ* are expected to lead to the identification of genes involved in controlling those phenotypes. In the present study, we screened suppressor mutations of complex colony morphology, strong invasive growth, and hyper-filamentous growth in the Σ1278b *hog1Δ/hog1Δ* background by constructing homozygous double mutants (i.e. *xxx::mTn/xxx::mTn hog1Δ/hog1Δ*).

**Figure 3:**
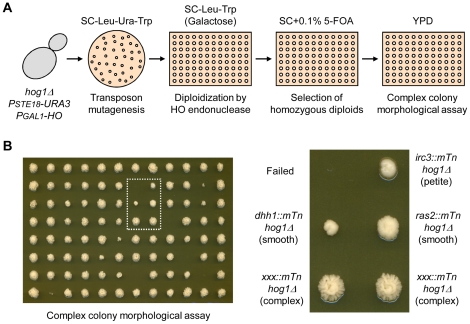
Screening suppressor mutations of the complex colony morphology or hyper-filamentous growth phenotype in the *hog1Δ/hog1Δ* backgrounds. (A) Strategy for screening the suppressor mutations. Using the haploid *hog1Δ PSTE18-URA3* strain carrying pJH283 (*PGAL1-HO::TRP1*) as a host strain, transposon insertion mutagenesis was performed and mutant strains defective in complex colony morphology were screened by visual inspection. The details are described in Materials and Methods. (B) One example of the screening results is shown. The candidates, smooth colony or less complex colony, were further analyzed: identification of the transposon insertion position, mating-type PCR, and morphological assay for invasive growth and filamentous growth.

Following the strategy shown in Figure 3A, a transposon insertion mutagenesis was performed using the haploid *hog1Δ PSTE18-URA3* strain carrying pJH283 (*PGAL1-HO*) as a host strain. Since the diploid *hog1Δ/hog1Δ* strain displays complex colony morphology even on YPD plates while the diploid wild-type strain does not [10], [16], mutant strains defective in formation of complex colony morphology were first screened by visual inspection (Figure 3B). From more than six thousand 5-FOA resistant strains (candidates for homozygous diploids) which were obtained at 93% success rate of randomly picked transformants, we isolated more than 100 mutant strains that showed smooth- or less complex-colony morphology. The diploid state in those was confirmed by PCR analysis of the mating-type locus. We amplified the transposon insertion regions of these strains by vectorette PCR and sequencing of the PCR products identified 49 unique genes (Table 1 and Table S1). Morphological developments (complex colony morphology, invasive growth, and filamentous growth) of all 49 homozygous double mutant strains on YPD plates were characterized, and the results are discussed below.

**Table 1:** Identified mutations that suppress enhanced morphological developments of the homozygous *hog1Δ/hog1Δ*γstrain.

Gene	CCM	IG	FG	Description of gene product	Reference
*ADE6*	– –	– –	–	Formylglycinamidine-ribonucleotide (FGAM)-synthetase	This study
*AMN1*	– –	–	–	Protein required for daughter cell separation, multiple mitotic checkpoints, and chromosome stability	[15]
*ATO2*	– –	– –	+	Putative transmembrane protein involved in export of ammonia	This study
*CLN1*	– –	–	– –	G1 cyclin involved in regulation of the cell cycle	[16], [23]
*DHH1*	– –	– –	–	Cytoplasmic DExD/H-box helicase	[24]
*FLO8*	– –	– –	– –	Transcription factor required for flocculation, diploid filamentous growth, and haploid invasive growth	[15], [20]
*GCN2*	– –	–	+	Protein kinase that phosphorylates the alpha-subunit of translation initiation factor eIF2	[19]
*HIR2*	– –	–	–	Subunit of the HIR complex	[15]
*HIR3*	– –	–	–	Subunit of the HIR complex	This study
*IES1*	–	+	–	Subunit of the INO80 chromatin remodeling complex	This study
*IMP2′*	–	–	–	Transcriptional activator involved in maintenance of ion homeostasis and protection against DNA damage	This study
*KRE11*	–	–	+	Subunit of the TRAPP II (transport protein particle) complex	This study
*KSS1*	– –	– –	– –	Mitogen-activated protein kinase involved in filamentous growth and pheromone response	[21]
*MSN1*	–	– –	–	Transcriptional activator involved in invertase expression and invasive growth/pseudohyphal differentiation	[15], [26]
*MTC5*	– –	– –	– –	Subunit of the SEA (Seh1-associated) complex	This study
*RAD1*	– –	– –	–	Single-stranded DNA endonuclease	This study
*RAD24*	–	–	+	Checkpoint protein involved in the activation of the DNA damage and meiotic pachytene checkpoints	This study
*RAS2*	– –	– –	– –	GTP-binding protein that regulates the nitrogen starvation response, sporulation, and filamentous growth	[15], [16]
*RDI1*	–	+	–	Rho GDP dissociation inhibitor involved in the localization and regulation of Cdc42	[22]
*RIM9*	– –	– –	– –	Protein of unknown function involved in the proteolytic activation of Rim101p in response to alkaline pH	[15], [27]
*SHE10*	– –	–	+	Putative glycosylphosphatidylinositol (GPI)-anchored protein of unknown function	This study
*SIR3*	– –	– –	–	Silencing protein that interacts with Sir2p and Sir4p, and histone H3 and H4 tails	This study
*SLP1*	–	–	–	Member of the SUN-like family of proteins	This study
*SPT2*	–	– –	–	Protein involved in negative regulation of transcription	This study
*STE7*	– –	– –	– –	MAPK kinase involved in pheromone response and pseudohyphal/invasive growth	[16], [21]
*TCO89*	–	–	–	Subunit of TORC1, a complex that regulates growth in response to nutrient availability	This study
*TEC1*	– –	– –	– –	Transcription factor required for haploid invasive and diploid pseudohyphal growth (TEA/ATTS family)	[14], [15], [16]
*UBP6*	–	–	–	Ubiquitin-specific protease	This study
*YDR306C*	– –	– –	+	F-box protein of unknown function	This study
*YHR177W*	– –	– –	– –	Putative protein of unknown function	This study

CCM: complex colony morphology, IG: invasive growth, FG: filamentous growth.

– –: severe defect, –: intermediate defect, +: similar to control (*hog1Δ/hog1Δ*).

### Mitochondrial Function Is Essential for Complex Colony Morphology

Sixteen of the 49 strains displayed petite and smooth colony morphology (Figure S2) and were unable to grow on plates containing glycerol as a sole carbon source (data not shown). These phenotypes suggest impaired respiratory growth, and all of these mutations are indeed linked to mitochondrial functions (Table S1). We confirmed that a *rho* ^0^ mutant (lacking mitochondria DNA) in the *hog1Δ/hog1Δ* background also displays petite and smooth colony morphology (Figure S2). Moreover, we identified three additional genes encoding proteins related to mitochondrial functions, *ILM1*, *SCO1*, and *SDH1* (Table S1). These results indicate that mitochondrial function is essential for complex colony morphology. Jin et al. have previously shown that mitochondrial dysfunction inhibits filamentous growth through the retrograde signaling pathway, which is a mitochondria-to-nucleus pathway transducing changes in mitochondrial function to specific adaptive changes in nuclear gene expression [15]. *ILM1* is known to be required for both mitochondrial function and slowed DNA synthesis-induced filamentous growth [17]. However, all of the mitochondria-related mutants identified by our screen poorly suppressed hyper-filamentous growth and strong invasive growth of the *hog1Δ/hog1Δ* strain (data not shown), suggesting that the hyper-filamentous phenotype of *HOG1* deletion involves multiple mechanisms that are not simply suppressed by mitochondrial dysfunction. Alonso-Monge et al. have recently reported that the *Candida albicans hog1* mutant shows an enhanced basal respiratory rate compared to the wild-type strain and suggested a link between Hog1 and mitochondrial function [18]. Therefore, it would be interesting to investigate whether and how Hog1 activated by hyperosmotic stress inhibits mitochondrial function during filamentous growth.

### Multiple Mechanisms Are Necessary for the Enhanced Morphological Developments of *hog1Δ/hog1Δ*

**Figure 4:**
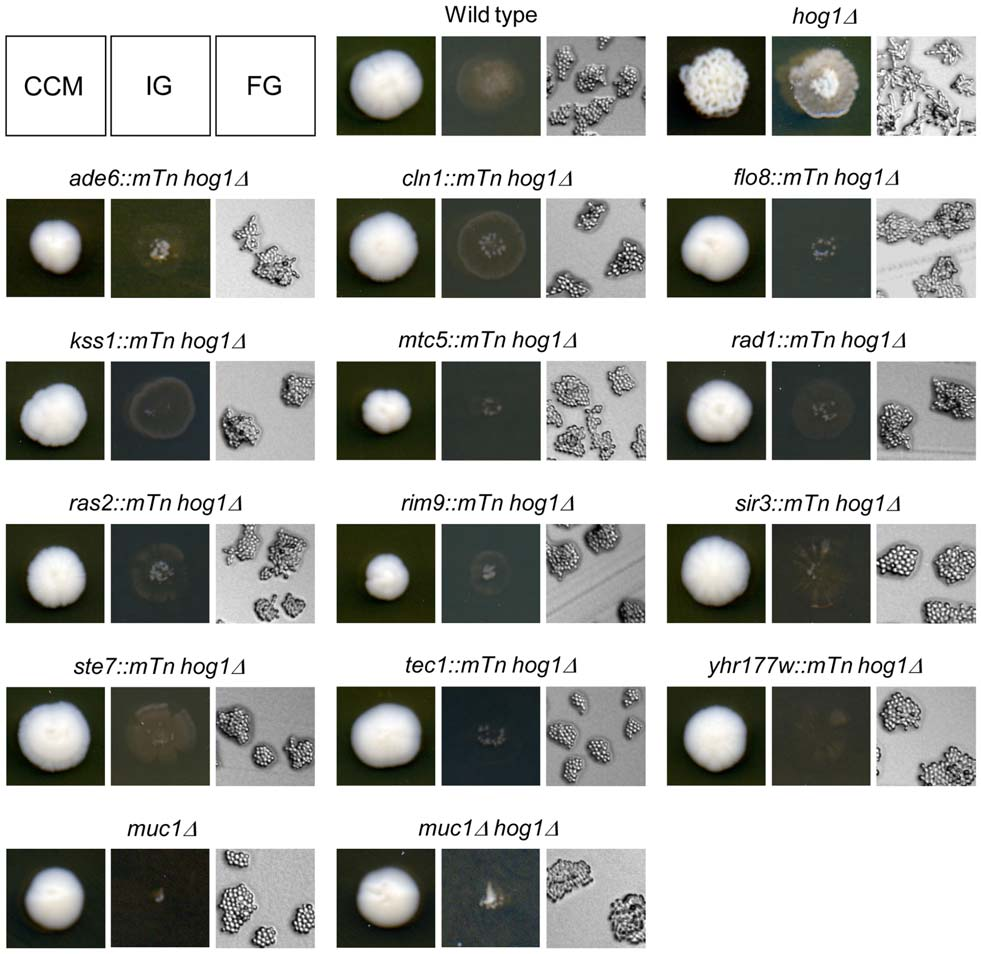
Morphological assay of homozygous double mutant strains which suppress enhanced morphological developments of *hog1Δ/hog1Δ*. CCM: complex colony morphology, IG: invasive growth, FG: filamentous growth. All other suppressor mutants identified are shown in Table 1.

In addition to the mitochondria-related mutations, we identified 30 mutations that suppress at least two of the enhanced morphological developments of *hog1Δ/hog1Δ* (Table S1) and representative mutants are shown in Figure 4. Thirteen of the 30 genes have previously been reported to be involved in at least one of the three morphological developments in the *S. cerevisiae* Σ1278b background [14], [15], [16], [19], [20], [21], [22], [23], [24]. Those genes include the well-known *STE7*, *KSS1*, *TEC1*, *RAS2*, and *FLO8* that regulate filamentous growth via the MAPK or cAMP-PKA signaling pathway. These two signaling pathways converge on the regulation of the *MUC1* (also known as *FLO11*) gene [25], which encodes a GPI-anchored cell surface mucin required for morphological developments. *DHH1* is involved in translational regulation of the Ste12 transcription factor which is regulated under the Kss1 MAPK pathway and essential for *MUC1* expression [24]. *GCN2* (general amino acid control system) and *MSN1* (transcriptional activator) are involved in the regulation of *MUC1* under certain nutrient conditions [19], [26]. Presumably, *RIM9* is also important for the regulation of *MUC1* through the pH-responsive Dfg16-Rim101 pathway [27]. Thus, our screen implies that impaired *MUC1* expression is sufficient to suppress enhanced morphological developments of *hog1Δ/hog1Δ*. Indeed, deletion of the *MUC1* gene in the *hog1Δ/hog1Δ* background lost the enhanced morphological developments and resulted in morphology similar to a *muc1Δ/muc1Δ* strain (Figure 4).

Seventeen of the 30 mutations occurred in genes not previously implicated in filamentous growth in *S. cerevisiae*. These gene products are involved in various cellular mechanisms, especially DNA damage checkpoint control (*IMP2′*, *RAD1*, and *RAD24*), gene expression via chromatin remodeling or histone-nucleosome assembly (*HIR2*, *HIR3*, *IES1*, *SIR3*, and *SPT2*), control of cell cycle or cell division (*AMN1* and *YHR177W*), and other functions. Although the present study did not reveal the morphological suppressing mechanism by deletion of these genes or physical interactions between Hog1 and the identified targets, our screen could highlight the genetic networks that are required for the enhanced morphological developments independent of the filamentous growth signaling pathways. Since the active Hog1 (Hog1-D170A/F318S) strain inhibits all of the morphological developments in a hyperosmotic stress-independent manner [10], the mechanisms identified in our screen might be negatively regulated by Hog1. While crosstalk between the HOG and filamentous growth MAPK pathways is well established [28], the present mutant collection will enable further analyses of connections between the HOG pathway and other mechanisms. Such efforts can contribute to understanding mechanisms of filamentous growth common between model yeasts and pathogenic yeasts as well as devising novel antifungal targets.

In conclusion, we demonstrate that combinatorial use of the *PGAL1-HO* and *PSTE18-URA3* genes is effective for construction of homozygous diploid strains and a useful tool when combined with large-scale screening. Genetic information of *S. cerevisiae* homozygous diploids obtained by the method proposed in this study may be useful for the studies of other organisms such as diploid *Candida albicans* in which construction of homozygous mutant strains is a difficult task.

## Materials and Methods

### Yeast Strains and Plasmids

The yeast strains used in this study are listed in Table 2. Standard yeast manipulations were performed as described previously [29]. To generate the *PSTE18-URA3* strains, a PCR product of *PSTE18-URA3* amplified from pPSTE18-URA3 (see below) using primers (AATGTGGCTGTGGTTTCAGGGTCCATAAAGCTTTTCAATTCATCTTTTTTCGTTGGCCGATTCATTCCCGAATTGGG and CATGCATTTAGAGCTCATACAGTTTTTTAGTTTTGC) was integrated into the *ura3-52* locus. Correct integration was verified by PCR using the *URA3* flanking primers (GGTGAAGGATAAGTTTTGACCATCAAAGAAGG and CGACCGAGATTCCCGGGTAATAACTG). To construct pPSTE18-URA3, a *STE18* promoter region (from –630 to +6) was amplified by PCR using primers (ATGCTGGATGAAGCTTAGTGTGATCTGATGTTCC and GAGAGTTTTGGGATCCTGTCATTCTTAGAATTATTG), and inserted into the HindIII-BamHI sites of pPAQY2-URA3 [10], resulting in replacement of the *AQY2* promoter by the *STE18* promoter. To generate the homozygous *PSTE18-URA3/PSTE18-URA3* strains, the haploid *PSTE18-URA3* strains (*MAT* **a** and *MAT* **α**) were crossed, and then (hyper-)filamentous strains were isolated on synthetic low ammonia dextrose (SLAD; 2% glucose, 50 µM ammonium sulfate, 0.17% yeast nitrogen base without amino acids/ammonium sulfate, supplemented with amino acids to satisfy nutritional requirements) plate. To generate *rho* ^0^ mutant strains, the YSH2381 and YSH2386 strains were grown on YPD plate containing 10 µg/ml ethidium bromide for 3 days. The resulting respiratory deficiency was confirmed by complete lack of growth on YPGlycerol (1% yeast extract, 2% peptone and 2% glycerol) plate. pJH283 (original name pFH800 [30]; *CEN*, *TRP1*, *PGAL1-HO*) was used to induce mating type switch of haploids.

**Table 2:** Yeast strains used in this study.

Strain	Genotype	Source
10560-6B	*MAT*α *leu2::hisG trp1::hisG his3::hisG ura3-52*	Lab collection
10560-4A	*MAT*a *leu2::hisG trp1::hisG his3::hisG ura3-52*	Lab collection
YSH1772	*MAT*α *leu2::hisG trp1::hisG his3::hisG ura3-52 hog1::kanMX4*	[10]
YSH2049	*MAT*a *leu2::hisG trp1::hisG his3::hisG ura3-52 hog1::kanMX4*	[10]
YSH2377	*MAT*α *leu2::hisG trp1::hisG his3::hisG ura3-52::PSTE18-URA3*	This study
YSH2379	*MAT*a *leu2::hisG trp1::hisG his3::hisG ura3-52::PSTE18-URA3*	This study
YSH2381	*MAT*a/α *leu2::hisG/leu2::hisG trp1::hisG/trp1::hisG his3::hisG/his3::hisG ura3-52::PSTE18-URA3/ura3-52::PSTE18-URA3*	This study
YSH2382	*MAT*α *leu2::hisG trp1::hisG his3::hisG ura3-52::PSTE18-URA3 hog1::kanMX*	This study
YSH2384	*MAT*a *leu2::hisG trp1::hisG his3::hisG ura3-52::PSTE18-URA3 hog1::kanMX*	This study
YSH2386	*MAT*a/α *leu2::hisG/leu2::hisG trp1::hisG/trp1::hisG his3::hisG/his3::hisG ura3-52::PSTE18-URA3/ura3-52::PSTE18-URA3 hog1::kanMX4/hog1::kanMX4*	This study
YSH2443	*MAT*a/α *leu2::hisG/leu2::hisG trp1::hisG/trp1::hisG his3::hisG/his3::hisG ura3-52::PSTE18-URA3/ura3-52::PSTE18-URA3 rho* ^0^	This study
YSH2445	*MAT*a/α *leu2::hisG/leu2::hisG trp1::hisG/trp1::hisG his3::hisG/his3::hisG ura3-52::PSTE18-URA3/ura3-52::PSTE18-URA3 hog1::kanMX4/hog1::kanMX4 rho* ^0^	This study
YSH2447	*MAT*a *leu2::hisG trp1::hisG his3::hisG ura3-52::PSTE18-URA3 hog1::LEU2*	This study
YSH2449	*MAT*a/α *leu2::hisG/leu2::hisG trp1::hisG/trp1::hisG his3::hisG/his3::hisG ura3-52::PSTE18-URA3/ura3-52::PSTE18-URA3 muc1::kanMX/muc1::kanMX*	This study
YSH2450	*MAT*a/α *leu2::hisG/leu2::hisG trp1::hisG/trp1::hisG his3::hisG/his3::hisG ura3-52::PSTE18-URA3/ura3-52::PSTE18-URA3 muc1::kanMX/muc1::kanMX hog1::LEU2/hog1::LEU2*	This study

### Transposon Insertion Mutagenesis

Mutant screening was performed using a genomic library mutagenized by random insertion of the transposon mTn-*lacZ/LEU2* [31]. The genomic library was digested by NotI, and the resulting DNA fragments were transformed into the haploid *hog1Δ PSTE18-URA3* strain carrying pJH283, and transformants were selected on synthetic complete (SC) plate lacking Leu, Ura, and Trp. The transformants were transferred on 1% galactose plates using a toothpick, and incubated overnight to induce diploidization. The cells were replicated on plates containing 0.1% 5-FOA using a 96-pin replicator (Singer RoToR) to select only diploids. Next, the 5-FOA resistant cells were replicated on YPD (1% yeast extract, 2% peptone and 2% glucose) plates and grown for 2 days at 30°C and for additional 5 days at room temperature. Suppressor mutant strains that showed no (smooth) or less complex colony morphology were screened by visual inspection.

### Characterization of Suppressor Mutant Strains

To determine the sites of transposon insertion in the isolated mutants, vectorette PCR was performed following the manual of the Yale Genome Analysis Center (http://ygac.med.yale.edu/). The purified PCR products were sequenced by Eurofins MWG Operon (Germany). To confirm diploidization of the mutant strains, mating-type was determined by PCR using three primers (AGTCACATCAAGATCGTTTATGG, GCACGGAATATGGGACTACTTCG, and ACTCCACTTCAAGTAAGAGTTTG) as described previously [32]. For colony morphological assay, yeast cells were grown on YPD plates for 2 days at 30°C and for additional 5 days at room temperature. For invasive growth assay, yeast cells (obtained for colony morphological assay) were washed off under flowing water and rubbed with a wet finger to remove cells that did not invade the agar. For filamentous growth assay, yeast cells were streaked on YPD plates and grown for 16 hours at 30°C, and the cells were visualized by light microscopy. To verify that the observed phenotype is due to the transposon insertion, homozygous double mutant strains were created again by deleting genes with a *kanMX* marker in the YSH2447 background carrying pJH283.

## Supporting Information

Figure S1 **Confirmation of diploidized yeast strains by mating-type PCR.** The mating-type PCR of *MAT* **a**, *MAT* **α**, and *MAT* **a**/**α** (diploid) cells provides 544-bp, 404-bp, and both PCR products, respectively. All of the 5-FOA resistant single colonies which were generated from the indicated haploid *PSTE18-URA3* strains carrying pJH283 provided the diploid specific PCR pattern. M: 100-bp DNA ladder.
(TIF)

Figure S2 **Identified mitochondria-related mutations that suppress complex colony morphology of the homozygous** ***hog1Δ/hog1Δ*** **strain.** Cells were grown on YPD plates for 2 days at 30°C and for additional 5 days at room temperature. A *rho* ^0^ mutation in the *hog1Δ/hog1Δ* background resulted in the same phenotype as the identified mitochondria-related mutations.
(TIF)

Table S1 **Identified mitochondria-related mutations that suppress complex colony morphology of the homozygous** ***hog1Δ/hog1Δ*** **strain.**
(DOC)

## References

[1] Giaever G, Chu AM, Ni L, Connelly C, Riles L (2002). Functional profiling of the *Saccharomyces cerevisiae* genome.. Nature.

[2] Klar AJ (2010). The yeast mating-type switching mechanism: a memoir.. Genetics.

[3] Herskowitz I, Jensen RE (1991). Putting the *HO* gene to work: practical uses for mating-type switching.. Methods Enzymol.

[4] Drinnenberg IA, Weinberg DE, Xie KT, Mower JP, Wolfe KH (2009). RNAi in budding yeast.. Science.

[5] Deutschbauer AM, Jaramillo DF, Proctor M, Kumm J, Hillenmeyer ME (2005). Mechanisms of haploinsufficiency revealed by genome-wide profiling in yeast.. Genetics.

[6] Lo HJ, Köhler JR, DiDomenico B, Loebenberg D, Cacciapuoti A (1997). Nonfilamentous *C. albicans* mutants are avirulent.. Cell.

[7] Hohmann S, Krantz M, Nordlander B (2007). Yeast osmoregulation.. Methods Enzymol.

[8] Chen RE, Thorner J (2007). Function and regulation in MAPK signaling pathways: lessons learned from the yeast *Saccharomyces cerevisiae*.. Biochim Biophys Acta.

[9] O'Rourke SM, Herskowitz I (1998). The Hog1 MAPK prevents cross talk between the HOG and pheromone response MAPK pathways in *Saccharomyces cerevisiae*.. Genes Dev.

[10] Furukawa K, Sidoux-Walter F, Hohmann S (2009). Expression of the yeast aquaporin Aqy2 affects cell surface properties under the control of osmoregulatory and morphogenic signalling pathways.. Mol Microbiol.

[11] de Godoy LM, Olsen JV, Cox J, Nielsen ML, Hubner NC (2008). Comprehensive mass-spectrometry-based proteome quantification of haploid versus diploid yeast.. Nature.

[12] Johnston M, Davis RW (1984). Sequences that regulate the divergent *GAL1-GAL10* promoter in *Saccharomyces cerevisiae*.. Mol Cell Biol.

[13] Boeke JD, LaCroute F, Fink GR (1984). A positive selection for mutants lacking orotidine-5′-phosphate decarboxylase activity in yeast: 5-fluoro-orotic acid resistance.. Mol Gen Genet.

[14] Mösch HU, Fink GR (1997). Dissection of filamentous growth by transposon mutagenesis in *Saccharomyces cerevisiae*.. Genetics.

[15] Jin R, Dobry CJ, McCown PJ, Kumar A (2008). Large-scale analysis of yeast filamentous growth by systematic gene disruption and overexpression.. Mol Biol Cell.

[16] Granek JA, Magwene PM (2010). Environmental and genetic determinants of colony morphology in yeast.. PLoS Genet.

[17] Kang CM, Jiang YW (2005). Genome-wide survey of non-essential genes required for slowed DNA synthesis-induced filamentous growth in yeast.. Yeast.

[18] Alonso-Monge R, Carvaihlo S, Nombela C, Rial E, Pla J (2009). The Hog1 MAP kinase controls respiratory metabolism in the fungal pathogen *Candida albicans*.. Microbiology.

[19] Braus GH, Grundmann O, Brückner S, Mösch HU (2003). Amino acid starvation and Gcn4p regulate adhesive growth and *FLO11* gene expression in *Saccharomyces cerevisiae*.. Mol Biol Cell.

[20] Liu H, Styles CA, Fink GR (1996). *Saccharomyces cerevisiae* S288C has a mutation in *FLO8*, a gene required for filamentous growth.. Genetics.

[21] Roberts RL, Fink GR (1994). Elements of a single MAP kinase cascade in *Saccharomyces cerevisiae* mediate two developmental programs in the same cell type: mating and invasive growth.. Genes Dev.

[22] Tiedje C, Sakwa I, Just U, Höfken T (2008). The Rho GDI Rdi1 regulates Rho GTPases by distinct mechanisms.. Mol Biol Cell.

[23] Loeb JD, Kerentseva TA, Pan T, Sepulveda-Becerra M, Liu H (1999). *Saccharomyces cerevisiae* G1 cyclins are differentially involved in invasive and pseudohyphal growth independent of the filamentation mitogen-activated protein kinase pathway.. Genetics.

[24] Park YU, Hur H, Ka M, Kim J (2006). Identification of translational regulation target genes during filamentous growth in *Saccharomyces cerevisiae*: regulatory role of Caf20 and Dhh1.. Eukaryot Cell.

[25] Rupp S, Summers E, Lo HJ, Madhani H, Fink G (1999). MAP kinase and cAMP filamentation signaling pathways converge on the unusually large promoter of the yeast *FLO11* gene.. EMBO J.

[26] Gagiano M, van Dyk D, Bauer FF, Lambrechts MG, Pretorius IS (1999). Msn1p/Mss10p, Mss11p and Muc1p/Flo11p are part of a signal transduction pathway downstream of Mep2p regulating invasive growth and pseudohyphal differentiation in *Saccharomyces cerevisiae*.. Mol Microbiol.

[27] Li W, Mitchell AP (1997). Proteolytic activation of Rim1p, a positive regulator of yeast sporulation and invasive growth.. Genetics.

[28] Saito H (2010). Regulation of cross-talk in yeast MAPK signaling pathways.. Curr Opin Microbiol.

[29] Amberg DC, Burke DJ, Strathern JN (2005). Methods in Yeast Genetics: A Cold Spring Harbor Laboratory Course Manual..

[30] Nickoloff JA, Singer JD, Hoekstra MF, Heffron F (1989). Double-strand breaks stimulate alternative mechanisms of recombination repair.. J Mol Biol.

[31] Burns N, Grimwade B, Ross-Macdonald PB, Choi EY, Finberg K (1994). Large-scale analysis of gene expression, protein localization, and gene disruption in *Saccharomyces cerevisiae*.. Genes Dev.

[32] Huxley C, Green ED, Dunham I (1990). Rapid assessment of *S. cerevisiae* mating type by PCR.. Trends Genet.

