# Supplementary figures and images for: Efficient Construction of Homozygous Diploid Strains Identifies Genes Required for the Hyper-Filamentous Phenotype in Saccharomyces cerevisiae
Source: PLoS One. 2011 Oct 21;6(10):e26584. doi: 10.1371/journal.pone.0026584 (PMC3198790; doi:10.1371/journal.pone.0026584)

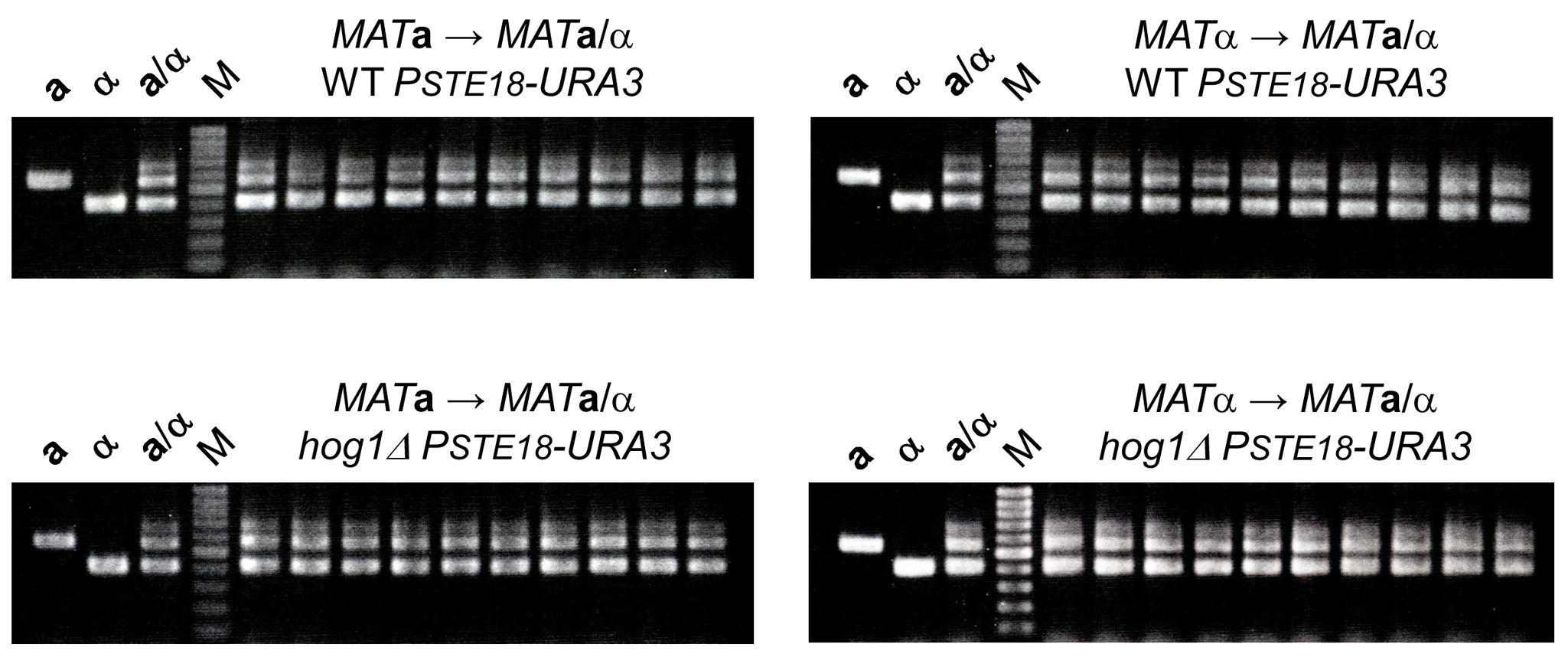

Supplement: Figure S1 — Confirmation of diploidized yeast strains by mating-type PCR. The mating-type PCR of MAT a, MAT α, and MAT a/α (diploid) cells provides 544-bp, 404-bp, and both PCR products, respectively. All of the 5-FOA resistant single colonies which were generated from the indicated haploid PSTE18-URA3 strains carrying pJH283 provided the diploid specific PCR pattern. M: 100-bp DNA ladder. (TIF) [file pone.0026584.s001.tif]

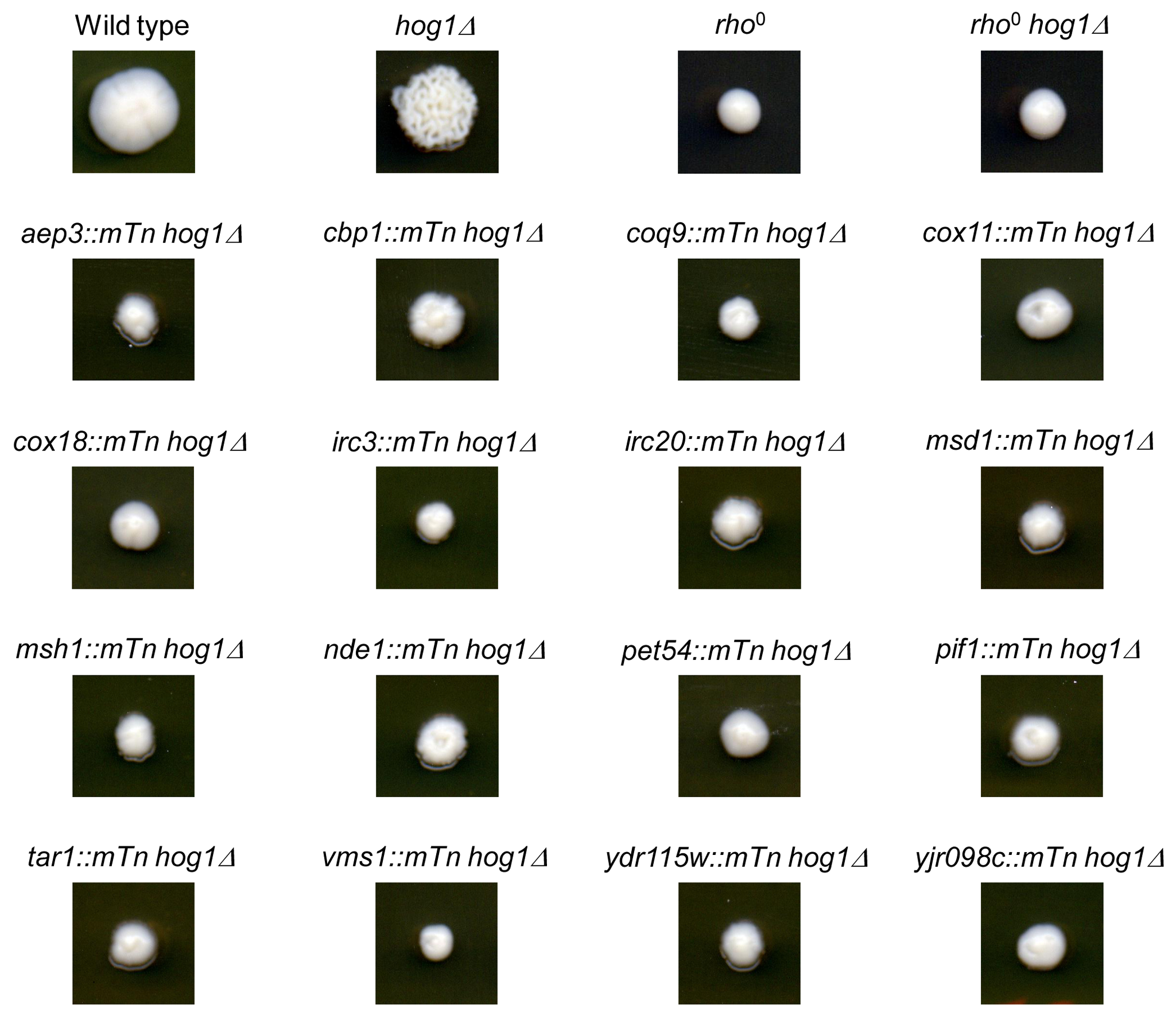

Supplement: Figure S2 — Identified mitochondria-related mutations that suppress complex colony morphology of the homozygous hog1Δ/hog1Δ strain. Cells were grown on YPD plates for 2 days at 30°C and for additional 5 days at room temperature. A rho 0 mutation in the hog1Δ/hog1Δ background resulted in the same phenotype as the identified mitochondria-related mutations. (TIF) [file pone.0026584.s002.tif]
