# Supplementary material for: Efficient Construction of Homozygous Diploid Strains Identifies Genes Required for the Hyper-Filamentous Phenotype in Saccharomyces cerevisiae
Source: PLoS One. 2011 Oct 21;6(10):e26584. doi: 10.1371/journal.pone.0026584 (PMC3198790; doi:10.1371/journal.pone.0026584)
Supplement: Table S1 — Identified mitochondria-related mutations that suppress complex colony morphology of the homozygous hog1Δ/hog1Δ strain. (DOC) [file pone.0026584.s003.doc]

**Table S1. Identified mitochondria-related mutations that suppress complex colony morphology of the homozygous *hog1Δ/hog1Δ*** **strain.**

**Gene Description of gene product**

*AEP3* Peripheral mitochondrial inner membrane protein, located on the matrix face of the membrane; stabilizes the bicistronic *AAP1-ATP6* mRNA encoding subunits 6 and 8 of the ATP synthase complex

*CBP1* Mitochondrial protein that interacts with the 5'-untranslated region of the COB mRNA and has a role in its stability and translation; found in a complex at the inner membrane along with Pet309p

*COQ9* Protein required for ubiquinone (coenzyme Q) biosynthesis and respiratory growth; localizes to the matrix face of the mitochondrial inner membrane in a large complex with ubiquinone biosynthetic enzymes

*COX11* Mitochondrial inner membrane protein required for delivery of copper to the Cox1 subunit of cytochrome c oxidase; association with mitochondrial ribosomes suggests that copper delivery may occur during translation of Cox1

*COX18* Mitochondrial integral inner membrane protein required for membrane insertion of C-terminus of Cox2; interacts genetically and physically with Mss2 and Pnt1; similar to *S. cerevisiae* Oxa1, *N. crassa* Oxa2, and *E. coli* YidC

*ILM1*  Protein of unknown function; may be involved in mitochondrial DNA maintenance; required for slowed DNA synthesis-induced filamentous

growth

*IRC3* Putative RNA helicase of the DEAH/D-box family; null mutant displays increased levels of spontaneous Rad52 foci; green fluorescent protein (GFP)-fusion protein localizes to the mitochondrion

*IRC20* Putative helicase; localizes to the mitochondrion and the nucleus; YLR247C is not an essential gene; null mutant displays increased levels of

spontaneous Rad52p foci

*MSD1* Mitochondrial aspartyl-tRNA synthetase, required for acylation of aspartyl-tRNA; yeast and bacterial aspartyl-, asparaginyl-, and lysyl-tRNA synthetases contain regions with high sequence similarity, suggesting a common ancestral gene

*MSH1* DNA-binding protein of the mitochondria involved in repair of mitochondrial DNA, has ATPase activity and binds to DNA mismatches; has homology to *E. coli* MutS; transcription is induced during meiosis

*NDE1* Mitochondrial external NADH dehydrogenase, a type II NAD(P)H:quinone oxidoreductase that catalyzes the oxidation of cytosolic NADH; Nde1 and Nde2 provide cytosolic NADH to the mitochondrial respiratory chain

*PET54* Mitochondrial inner membrane protein that binds to the 5' UTR of the *COX3* mRNA to activate its translation together with Pet122 and Pet494; also binds to the *COX1* Group I intron AI5 beta to facilitate exon ligation during splicing

*PIF1* DNA helicase; exists in two forms; the nuclear form is involved in telomere formation and elongation; acts as a catalytic inhibitor of telomerase; the mitochondrial form is involved in repair and recombination of mitochondrial DNA

*SCO1* Copper-binding protein of the mitochondrial inner membrane, required for cytochrome c oxidase activity and respiration; may function to deliver copper to cytochrome c oxidase; has similarity to thioredoxins

*SDH1* Flavoprotein subunit of succinate dehydrogenase (Sdh1p, Sdh2p, Sdh3p, Sdh4p), which couples the oxidation of succinate to the transfer of

electrons to ubiquinone as part of the TCA cycle and the mitochondrial respiratory chain

*TAR1* Mitochondrial protein potentially involved in regulation of respiratory metabolism; interacts genetically with *RPO41* and physically with Coq5p;

encoded within the 25S rRNA gene on the opposite strand

*VMS1* Zinc finger protein, forms a mitochondrially-associated complex with Cdc48p and Npl4p under oxidative stress that is required for ubiquitin-

mediated mitochondria-associated protein degradation (MAD); conserved in *C. elegans* and human

*YDR115W* Putative mitochondrial ribosomal protein of the large subunit, has similarity to *E. coli* L34 ribosomal protein; required for respiratory growth, as are most mitochondrial ribosomal proteins

*YJR098C* Putative protein of unknown function; the authentic, non-tagged protein is detected in highly purified mitochondria in high-throughput studies
